# The Impact of a Preoperative Cognitive Behavioural Therapy (CBT) on Dysfunctional Eating Behaviours, Affective Symptoms and Body Weight 1 Year after Bariatric Surgery: A Randomised Controlled Trial

**DOI:** 10.1007/s11695-015-1673-z

**Published:** 2015-04-19

**Authors:** Hege Gade, Oddgeir Friborg, Jan H. Rosenvinge, Milada Cvancarova Småstuen, Jøran Hjelmesæth

**Affiliations:** Morbid Obesity Centre, Vestfold Hospital Trust, P.O. Box. 2168, 3103 Tønsberg, Norway; Faculty of Health Sciences, Department of Psychology, UiT – The Arctic University of Norway, Tønsberg, Norway; Department of Endocrinology, Morbid Obesity and Preventive Medicine, Institute of Clinical Medicine, University of Oslo, Oslo, Norway

**Keywords:** Dysfunctional eating behaviours, Cognitive behavioural therapy, Anxiety, Depression, Bariatric surgery

## Abstract

**Background:**

To examine whether a preoperative cognitive behavioural therapy (CBT) intervention exceeds usual care in the improvements of dysfunctional eating behaviours, mood, affective symptoms and body weight 1 year after bariatric surgery.

**Methods:**

This is a 1-year follow-up of a single centre parallel-group randomised controlled trial (http://clinicaltrials.gov/ct2/show/NCT01403558). A total of 80 (55 females) patients mean (SD) age 44 (10) years were included. The intervention group received 10 weeks of CBT prior to bariatric surgery, and the control group received nutritional support and education. Both groups were assessed at baseline (T0), post CBT intervention/preoperatively (T1), and 1 year postoperatively (T2). Using a mixed modelling statistical approach, we examined if the CBT group improved more across time than the control group.

**Results:**

Our hypothesis was not supported as both groups had comparable improvements in all outcomes except for anxiety symptoms. Body weight declined by 30.2 % (37.3 kg) in the CBT group and by 31.2 % (40.0 kg) in the control group from baseline to follow-up, *p* = 0.82.

There were statistically significant reductions in anxiety and depression symptoms in the CBT group between T0 and T1 and between T1 and T2 for depression only. However, in the control group, the anxiety score did not change significantly. The CBT group showed an earlier onset of improvements in all eating behaviours and affective symptoms than the control group.

**Conclusion:**

The 10-week CBT intervention showed beneficial effects preoperatively, but the non-significant group differences postoperatively indicate a genuine effect of surgery.

## Introduction

In patients with extreme obesity undergoing bariatric surgery, there is a high rate of dysfunctional eating behaviours (DE) (i.e. emotional eating, uncontrolled eating and cognitive restraint) both prior to [1–5] and after surgery [5–7]. Gastric bypass, a common bariatric procedure, promotes weight loss mainly by reducing appetite, thereby helping the patient to change eating behaviours [8, 9]. Between 20 and 30 % of patients undergoing gastric bypass regain weight around 2 years after surgery [10, 11], and the notable individual differences in the amount of weight loss [12, 13] may be partly accounted for by sustained DE [14]. Data from the LABS-study [13] reporting 3 years change in weight show that the majority of patients reached their nadir weight 1 year after surgery. Additionally, five sub-groups with different patterns of weight loss were identified. These patterns showed a variability of weight changes starting at 6 months postoperatively. Cognitive restraint [15] and emotional eating [16] before surgery have been identified as predictors for postoperative body weight. Thus, whilst higher cognitive restraint may predict greater weight loss [15], increased emotional eating may predict suboptimal weight loss or weight regain [16]. Symptoms of mood and anxiety are also highly prevalent in this patient population [5, 17–19]. Except for short-term improvements of mood and affective symptoms after surgery [17, 20], mood and anxiety *disorders* diagnosed preoperatively tend to remain unchanged long-term [17].

Psychological interventions may alleviate both these comorbidities and DE. Some studies [21, 22] show that such kind of preoperative interventions may reduce both psychological co-morbidity and DE. In the studies of Ashton et al. [21, 23], a brief cognitive behavioural therapy (CBT) intervention of 4 weeks reduced binge eating behaviours before surgery. Additionally, the patients who improved their eating behaviours lost significantly more weight 1 year after surgery than those who did not. Abiles et al. [22] showed that a 12 week group-based CBT intervention reduced psychological co-morbidity both in patients with or without binge eating disorder (BED). In the second stage of the preoperative intervention, the patients were offered weekly follow-ups including dietary counselling and were recommended to follow a 1500 kcal diet. More than half of the patients had a preoperative weight loss of ≥10 %.

Other studies have provided additional information on the effects of postoperative interventions. The results from a pilot study by Sarwer et al. [24] indicate that dietary counselling may have a short-term (6 months) effect on weight loss and eating behaviours. In contrast, a controlled study [25] did not lend support to the effect of unspecified psychological support, yet this study restricted the outcome variable to weight reduction only.

Less is known though, about the longer-term impact of pre- or postoperative behavioural interventions on DE, psychological co-morbidity and weight loss. One systematic review and meta-analysis of postoperative behavioural interventions [26] concluded that greater weight loss may be achieved. However, the validity of these findings is restricted by uncontrolled study designs, measurements and contents of interventions.

In the evaluation of the impact of such interventions, the temporal aspect of postoperative follow-up is of importance. One year after surgery may be considered “short-term”, as significant differences in outcomes of body weight and DE may be seen at later stages.

Using an RCT-design, we recently demonstrated [27] that compared with usual care, a 10-week preoperative CBT significantly improved DE as well as affective symptoms immediately before the time of surgery. Whether these beneficial effects are sustained or whether the preoperative intervention may give any *additional* positive effects beyond surgery remain unknown.

In the present study, we anticipated that the outcome variables change differently across time depending on the consultations offered (CBT vs usual care). The hypothesis was that a preoperative CBT intervention would perform better than usual care in reducing DE and body weight as well as mood and anxiety symptoms at a 1-year follow-up after surgery.

## Methods

### Trial Design and Setting

This is the second part (1-year follow up) of a single centre parallel-group randomised controlled trial (http://clinicaltrials.gov/ct2/show/NCT01403558) conducted at a tertiary care centre in Norway between September 2011 and December 2013 [2, 27].

The study design was mixed factorial. The within factor had three levels (a repeated time factor: T0, T1 and T2). All outcome variables were measured at baseline (T0), post CBT intervention (preoperatively) (T1), and 1 year postoperatively (T2). The between factor had two levels: the control (G0) and the intervention (G1) group. The two-way interaction (*Group* × *Time*) thus represented a test of the hypothesised between-group mean difference in the outcome variables over time.

### Participants

All recruited participants were accepted for bariatric surgery. Those who agreed to take part in the study were included after providing written informed consent, both at inclusion and at 1-year postoperative follow-up. Unlike in North America, a preoperative psychological evaluation is not standard practise in Norway. Therefore, no patients were excluded from the study based on a psychological evaluation. Of note, all patients who were invited to participate in the study were already accepted for bariatric surgery.

### Interventions

During the 4 months prior to surgery, patients in both treatment arms were offered up to three voluntary consultations from either a medical doctor, a dietician, a nurse or a physical therapist tailored to the patients’ individual needs. The CBT intervention has been described in more detail elsewhere [28], but this 10-week treatment condition consisted of learning to recognise triggers of DE, i.e. identifying how automatic thoughts and dysfunctional cognitions, negative moods and overeating are interrelated. Moreover, weekly home-work tasks were used to break the DE-patterns which are a common problem for patients suffering from extreme obesity. Thus, the main purpose of the intervention was to improve self-monitoring and self-regulation of eating behaviour. Some individual adjustments during the course of therapy were allowed to accommodate for the fact that some patients spent more time working on obtaining more regularity in eating, whilst others addressed cognitive negative self-talk in order to reduce emotionally triggered eating behaviour.

In the year following the surgery, all patients were invited to attend one group session with a clinical nutritionist and another with a physical therapist. The patients were additionally offered two individual consultations with a physician.

### Covariates and Outcomes

The demographic variables at T0 comprised age, gender, educational level, and employment status. Body weight and height were measured with light clothing and no shoes using a digital scale (Soehnle Professional 2755, http://www.soehnle.de/) and a wall mounted stadiometer (Seca 240, http://www.stadiometer.com/), respectively. Body weight was measured at all three time points.

The clinical variables were collected at all-time points through a web-based solution (http://fluidsurveys.com/ and https://metreno.com/) and comprised the Three-Factor Eating Questionnaire (TFEQ R-21) [28, 29] measuring DE, and the Hospital Anxiety and Depression Scale (HADS) [30, 31] measuring symptoms of anxiety and depression, respectively.

The TFEQ R-21 has been validated for use in obese individual [28]. It consists of 21 items comprising three subscales, i.e. “Emotional eating” (EE; 6 items; Cronbach’s *α* = 0.92), “uncontrolled eating” (UE; 9 items; *α* = 0.81) and “cognitive restraint of eating” (CR; 6 items, *α* = 0.74). According to the manual, the three subscales were transformed to a 0–100 scale to become comparable [28]. Higher scores indicate higher levels of dysfunction. The reliability of the subscales in the present study was comparable to previous reports [28].

The HADS (30) is a self-report measure of anxiety and non-vegetative affective symptoms [30, 31]. Seven items assess depression (HADS-D) and seven items measure anxiety (HADS-A), respectively. Items are scored 0–3 yielding a range of 0–21 within each subscale. A cut-off ≥8 is used in Norway to indicate a clinically probable impairment due to depression or anxiety [32]. In the current study, the Cronbach’s alphas for HADS-A and HADS-D were 0.77 and 0.70, respectively.

### Statistical Methods

Continuous variables were described using means and standard deviations (SD), categorical data as counts and percentages. Crude differences between the groups were assessed with *t* tests (continuous data) and chi-square tests (categorical data). Linear mixed model regression analyses were used in order to estimate both random and fixed effects. Time, group and their interaction represented fixed effects, whilst individual differences at baseline were accounted for by a random intercept parameter. A diagonal covariance structure was specified to accommodate for heterogeneous residual variances across time. Restricted maximum likelihood estimation was used to produce unbiased estimates of the model parameters. All overall effects were analysed using the *F* tests. The results were presented as estimated means with 95 % confidence intervals (CI). Least significant difference (LSD) post hoc tests were used to compare selected means at given time points. Due to the exploratory nature of our study, we did not use any correction for multiple testing. *P* values <0.05 were considered statistically significant, and all tests were two-tailed. The software IBM SPSS version 17 (SPSS, Chicago, IL, USA) was used to conduct all analyses.

### Sample Size

The sample size was based on pre-estimations from the preoperative intervention. According to clinical experience, reductions in the emotional and uncontrolled eating scores of 15 % or more are considered to be clinically meaningful. A conservative estimate was that no patients in the control group, and at least 30 % in the intervention group, would achieve this treatment goal before surgery. Given this difference between the treatment groups, a 90 % statistical power, a significance level of 5 % and a dropout rate of 40 %, a minimum sample size of 80 patients was required at baseline. To allow for a 20 % withdrawal rate, we included 102 patients at baseline, and 83 patients completed all assessments 1 year after surgery [27].

### Randomisation

A block randomisation procedure (http://www.randomizer.org) was employed (with blocks of 4) to ensure balance between the groups. Two research assistants at the treatment centre with no affiliation to the study had access to the randomisation file. After having read and signed the informed consent letter and completed the baseline measurements, the patients as well as the first author were informed about the allocated treatment arm. The allocation ratio was 1:1 [27].

## Results

### Recruitment and Participant Flow

**Fig. 1:**
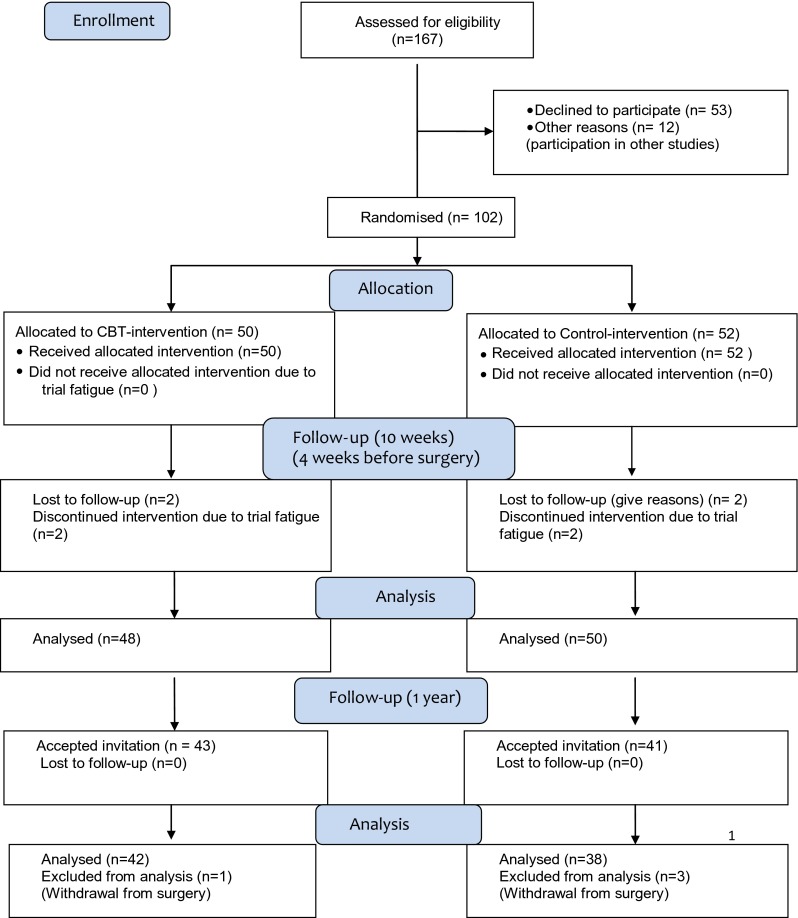
Participant flow: Patients with extreme obesity assessed for eligibility, randomisation and follow-up

Eighty-four patients accepted to participate at follow-up, but as four patients refused surgery, the final sample included 80 patients (Fig. 1). The majority of patients (69 %) were female, and the mean (SD) BMI was at T0 43.7 (4.9) kg/m^2^. There were no statistically significant differences between the groups regarding gender, BMI or level of education (Table 1). Eighty-six percent in the intervention group and 82 % in the control group underwent Roux-en-Y gastric bypass. The remaining patients underwent sleeve gastrectomy.

**Table 1:** Baseline demographics amongst 80 patients who underwent bariatric surgery by treatment arm

	Total (*n* = 80)	Intervention (*n* = 42)	Controls (*n* = 38)	*p* values
BMI (kg/m^2^)	43.7 (4.9)	43.6 (5.1)	43.5 (4.7)	0.742
Weight (kg)	128.7 (18.1)	129.5 (17.2)	127.7 (19.2)	0.661
Gender				
Female	55	27	28	0.369
Male	25	15	10	
Age (years)	44.3 (10)	44.1 (9.8)	41.2 (9.6)	0.152
Educational level				
12th grade	66 (82.5)	34 (81.0)	32 (84.2)	0.705
High school/college degree	14 (17.5)	8 (19)	6 (15.8)	
Employment				
Employed	45 (56.3)	22 (52.4)	23 (60.5)	0.671
Unemployed	4 (5)	3 (67.1)	1 (2.6)	
Temporary pension	17 (21.3)	9(21.4)	8 (21.1)	
Disabled	14 (17.5)	8 (19.0)	6 (15.8)	

Data presented as observed mean (SD) or number (%)

### Analyses of Treatment Effects

Neither of the groups had any significant change in body weight from T0 to T1 (data not shown). In contrast, body weight declined significantly after surgery (T1-T2) in both the CBT and control group; *M*_diff_ (95 % CI) = −37.3 (−40.4 to −34.2) kg and −40.0 (−43.3 to −36.7) kg, respectively, both *p*’s < 0.001. To summarise, in the CBT and the control group, the body weight was reduced by 30.9 and 31.2 %, respectively, from baseline to 1-year follow-up (*p* = 0.816).

**Fig. 2:**
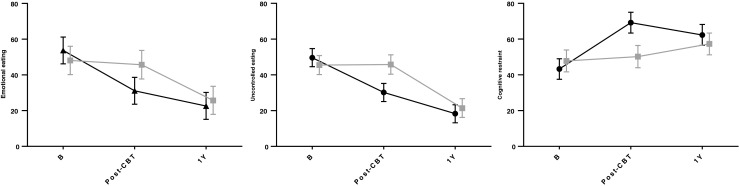
Changes in the three facets of dysfunctional eating behaviours (DE) by treatment arm measured by the TFEQ R-21: emotional eating, uncontrolled eating and cognitive restraint. *B* baseline (4 months before surgery), *post-CBT* after CBT intervention and before surgery, *1y* 1 year after surgery. Values presented as estimated means with 95 % CI from linear mixed-effects models. High scores represent more emotional eating, uncontrolled eating or cognitive restraint. CBT group in black (*filled triangles*) and control group in grey (*filled squares*)

**Fig. 3:**
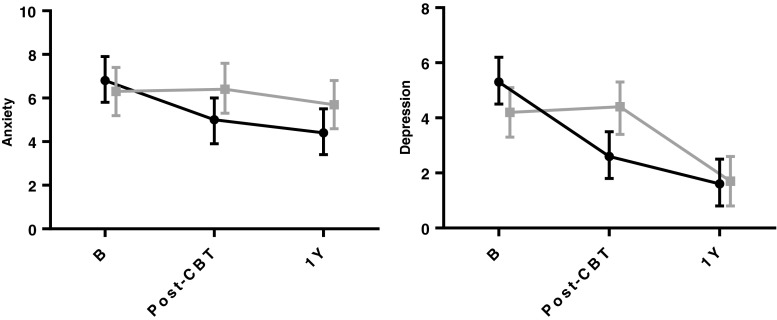
Changes in symptoms of anxiety and depression by treatment arm measured by the HADS. *B* baseline (4 months prior to surgery), *post-CBT* after CBT intervention and before surgery, *1y* 1 year after surgery. Values presented as estimated means with 95 % CI from linear mixed-effects models. High scores represent more anxiety or depression. CBT group in black (*filled triangles*) and control group in grey (*filled squares*)

The unadjusted means for all other outcome variables are presented in Table 2. The differential change in eating behaviours and affective symptoms across time and groups are presented in Figs. 2 and 3. The main effect of *time* was significant (*p* < 0.001) indicating an improvement in both groups across time (from T0 to T2). The interaction *group* × *time* was statistically significant for all outcome variables (all *p*’s < 0.01), except BMI, thus indicating that the improvement occurred at different time points in the two groups. Follow-up post hoc tests were therefore needed to pin-point which *group* differences were present at T1 and T2. There were no statistically significant differences in changes in body weight and DE between the patients who underwent RYGB and GS (all *p* values above).

**Table 2:** Treatment effects across time by treatment arm

Outcomes	EE		UE		CR		Anxiety		Depression	
	CBT	Control	CBT	Control	CBT	Control	CBT	Control	CBT	Control
	M 95 % CI	M 95 % CI	M 95 % CI	M 95 % CI	M 95 % CI	M 95 % CI	M 95 % CI	M 95 % CI	M 95 % CI	M 95 % CI
Baseline	53.7	48.1	49.6	45.5	43.3	47.8	6.8	6.3	5.3	4.2
	46.2–61.2	40.2–56.0	44.6–54.7	40.2–50.8	37.5–49.0	41.7–53.8	5.7–7.9	5.2–7.4	4.5–6.2	3.3–5.1
Post-intervention	31.1	45.7	30.2	45.8	69.2	50.2	5.0	6.4	2.6	4.4
	23.6–38.6	37.7–53.7	25.1–35.2	40.4–51.2	63.4–75.0	44.0–56.4	3.9–6.0	5.3–7.6	1.8–3.5	3.4–5.3
Follow-up	22.6	25.7	18.3	21.4	62.3	57.3	4.4	5.7	1.6	1.7
	15.1–30.2	17.8–33.6	13.2–23.3	16.2–26.7	56.5–68.2	51.2–63.4	3.4–5.5	4.6–6.8	0.7–2.5	0.8–2.6

Data presented as unadjusted means with confidential intervals (CI). EE (emotional eating), UE (uncontrolled eating) and CR (cognitive restraint) were measured by the Three-Factor Eating Questionnaire (TFEQ R-21), and symptoms of anxiety and depression were measured by the Hospital Anxiety and Depression Scale (HADS)

### Post Hoc Tests

#### Dysfunctional Eating

Pairwise post hoc tests were used to examine mean differences (*M*_diff_) between time (T0-T1-T2) and groups (CBT vs control). They revealed significant improvements in EE, UE and CR in the CBT group between T0 and T1; *M*_diff_ (95 % CI) = −22.6 (−30.3 to −14.9), −19.5 (−26.9 to −12.1) and 25.9 (17.4 to 34.5), respectively, all *p*’s < 0.001. Further improvements between T1 and T2 were evident for EE and UE only; *M*_diff_ = −8.5 (−17.2 to −0.3), *p* = 0.02 and −11.9 (−18.4 to −5.4), *p* < 0.001, respectively.

The improvement in the control group was only evident between T1 and T2 for EE and UE; *M*_diff_ = −20.0 (−29.2 to −10.9) and −24.3 (−31.2 to −17.4), both *p’s* < 0.001, and between T0 and T2 for CR; *M*_diff_ = 9.5 (0.1 to 19.1), *p* = 0.02.

Group differences: the post hoc tests revealed significant group differences favouring CBT only at T1 for EE, UE and CR; *M*_diff_ = −14.6 (−25.1 to −4.1), −15.6 (−22.9 to −8.2), −19.0 (−27.0 to −10.9), all *p’s* < 0.001, respectively, but not at T2.

#### Anxiety and Depression

There was a significant reduction in anxiety and depression symptoms in the CBT group between T0 and T1; *M*_diff_ = −1.9 (−3.1 to −0.7), and −2.7 (−4.1 to −1.3), respectively, both *p*’s < 0.001, and was borderline significant between T1 and T2 for depression; *M*_diff_ = −1.0 (−2.2 to 0.1), *p* = 0.08. In the control group, the anxiety score did not change significantly, whereas depression scores went down significantly between T1 and T2; *M*_diff_ = −2.7 (−3.9 to −1.5), *p* < 0.001.

##### Group Differences

No significant group differences were observed for anxiety, whereas a significant group difference favouring CBT was evident at T1; *M*_diff_ = −1.7 (−3.1 to −0.4), *p* = 0.01, for depression, but not at T2.

## Discussion

Our hypothesis that CBT would improve dysfunctional eating patterns, mood and anxiety symptoms 1 year after surgery was not supported. Apart from a comparable weight loss, the two groups revealed different patterns of changes in all eating behaviours and affective symptoms during the follow-up time.

Treatment effects comparisons with other studies are difficult as no other studies have examined the impact of a CBT intervention versus a control group over time. Our findings do however concur with a pilot-study of Sarwer et al. [24], which showed that initial short-term effects on weight loss and eating behaviours after postoperative dietary counselling waned off after the first 4 months. As shown in Figs. 2 and 3, the patterns of change suggest that the benefit of the CBT intervention exceeded usual care *before* surgery. However, at the 1-year follow-up, the CBT treatment did not have any *additional* effects beyond the surgery on eating behaviours, affective symptoms or body weight. The CBT intervention thus exceeded usual care in terms of *an earlier onset* of reduction of DE and affective symptoms, which represents a beneficial improvement in mental health in terms of facilitating functional coping with daily stress as well as control over eating. These findings are in line with Abiles et al. [22] indicating that a preoperative CBT intervention may improve psychological co-morbidity. Indicated by the present, as well as by previous research [17, 18], depressive symptoms usually drop more than symptoms of anxiety following bariatric surgery.

With respect to the course, our findings concur with studies [8, 9, 12, 18] showing more positive enduring changes in DE, affective symptoms and body weight, and that the surgery itself had a comprehensive effect on DE by limiting the possibility of consuming large amounts of food. However, the effects of bariatric surgery on DE and affective symptoms seem to decrease over time [18]. It may be the case that the CBT intervention was not potent enough to maintain further improvements that exceeded usual care 1 year after bariatric surgery. The clinical value of the treatment may thus be limited, or at least indicating a need to address issues related to maintenance of effects more carefully in psychological treatments. On the other hand, as bariatric surgery had a comprehensive effect on all these outcomes, our expectations of CBT being superior to usual care 1 year after surgery might have been too optimistic. According to the findings of Courcoulas et al. [13], the majority of the patients reached their maximum weight loss 1 year postoperatively, a period that has been labelled “the honeymoon phase”. As the biological effects of the surgery have not yet started to wane, and the majority of the patients are at their nadir weight, it may be difficult to identify any additional psychological effects at this time. On the other hand, the LABS-study [13] showed that the variability in the weight loss trajectories increased from about 6 months, postoperatively. Considering the profound biological effects that surgery provides during the first year, a 1 year follow-up gap may be considered rather short-term. Hence, the CBT intervention may still exert an influence, particularly when the problems with maintaining the weight loss start.

A minor objection may relate to the lack of statistical power as the sample size was calculated to detect preoperative (T1) treatment effects. As Table 2 reports, there were differences between the groups in the hypothesised directions at T2 that a larger sample size might have deemed significant. These differences would on the other hand probably have minor clinical importance.

Our study had considerable strengths, notably the randomised controlled design and the unbiased selection of patients due to the consecutive recruitment procedure. Moreover, CBT covers a number of different approaches which differ in their emphasis on cognitive versus behavioural principles and techniques. In contrast to previous studies, we have [27] designed a treatment manual with a detailed outline of the intervention, which makes replications more accurate. A limitation may be noted for the 1 year follow-up time. Accordingly, a later follow-up assessment might clarify whether the effects on DE and body weight would become more prominent when the biological effects of the surgery wane.

In case of a clinical replication, common versus specific psychotherapy effects need to be sorted out by including more than one therapist as well as including measures of treatment alliance and therapist competence. Furthermore, future studies should consider including a control group having an equal number of attendance sessions, hence ruling out alternative explanations related to differences in dose–response and placebo. Future studies should also include a measure of binge eating symptoms, and address the issue of treatment potency in terms of content and time of delivery.

## Conclusions

Our results confirmed that eating behaviours, affective symptoms and body weight improve the first year after bariatric surgery. The preoperative CBT initiated a faster improvement in dysfunctional eating and affective symptoms, but it was no longer superior to usual care following surgery.
